# Expression of caspases 3, 6 and 8 is increased in parallel with apoptosis and histological aggressiveness of the breast lesion

**DOI:** 10.1038/sj.bjc.6690735

**Published:** 1999-10

**Authors:** M Vakkala, P Pääkkö, Y Soini

**Affiliations:** Department of Pathology, University of Oulu and Oulu University Hospital, PO Box 5000, Kajaanintie 520, Oulu, 90701, Finland

**Keywords:** apoptosis, caspase, breast, carcinoma

## Abstract

The aim of this investigation was to study the expression of caspases 3, 6 and 8 and their association to apoptosis in preneoplastic and neoplastic lesions of the breast. The material consisted of nine benign breast epithelial hyperplasias, 15 atypical hyperplasias, 74 in situ and 82 invasive carcinomas. The extent of apoptosis was assessed by the TUNEL method and caspase 3, 6 and 8 expression by immunohistochemistry with specific antibodies. Increased caspase 3 immunopositivity, as compared to staining of normal breast ductal epithelium, was seen in 22% of benign epithelial hyperplasias, 25% of atypical hyperplasias, 58% of in situ carcinomas and 90% of invasive carcinomas. The corresponding percentages for caspase 6 and 8 were 11%, 25%, 60%, 87% and 22%, 57%, 84%, 83% respectively. In high-grade in situ lesions there were significantly more cases with strong caspase 3, 6 and 8 immunoreactivity than in low- and intermediate-grade lesions (*P* = 0.0045, *P* = 0.049 and *P* = 0.0001 respectively). In invasive carcinomas, however, no association between a high tumour grade and caspase 3, 6 or 8 expression was found (*P* = 0.27, *P* = 0.26 and *P* = 0.69 respectively). The mean apoptotic index was 0.14 ± 0.14% in benign epithelial hyperplasias, 0.17 ± 0.12% in atypical hyperplasias, 0.61 ± 0.88% in in situ carcinomas and 0.94 ± 1.21% in invasive carcinomas. In all cases strong caspase 3, 6 and 8 positivity was significantly associated with the extent of apoptosis (*P* < 0.001, *P* = 0.015 and *P* = 0.050 respectively). The results show that synthesis of caspases 3, 6 and 8 is up-regulated in neoplastic breast epithelial cells in parallel to the increase in the apoptotic index and progression of the breast lesions. © 1999 Cancer Research Campaign


*British Journal of Cancer* (1999) **81**(4), 592–599
© 1999 Cancer Research Campaign
Article no. bjoc.1999.0735

### Expression of caspases 3, 6 and 8 is increased in parallel with apoptosis and histological aggressiveness of the breast lesion

**M Vakkala, P Pääkkö and Y Soini**

Department of Pathology, University of Oulu and Oulu University Hospital, PO Box 5000, Kajaanintie 520, 90701 Oulu, Finland

**Summary** The aim of this investigation was to study the expression of caspases 3, 6 and 8 and their association to apoptosis in preneoplastic and neoplastic lesions of the breast. The material consisted of nine benign breast epithelial hyperplasias, 15 atypical hyperplasias, 74 in situ and 82 invasive carcinomas. The extent of apoptosis was assessed by the TUNEL method and caspase 3, 6 and 8 expression by immunohistochemistry with specific antibodies. Increased caspase 3 immunopositivity, as compared to staining of normal breast ductal epithelium, was seen in 22% of benign epithelial hyperplasias, 25% of atypical hyperplasias, 58% of in situ carcinomas and 90% of invasive carcinomas. The corresponding percentages for caspase 6 and 8 were 11%, 25%, 60%, 87% and 22%, 57%, 84%, 83% respectively. In high-grade in situ lesions there were significantly more cases with strong caspase 3, 6 and 8 immunoreactivity than in low- and intermediate-grade lesions ($P = 0.0045$, $P = 0.049$ and $P = 0.0001$ respectively). In invasive carcinomas, however, no association between a high tumour grade and caspase 3, 6 or 8 expression was found ($P = 0.27$, $P = 0.26$ and $P = 0.69$ respectively). The mean apoptotic index was 0.14 ± 0.14% in benign epithelial hyperplasias, 0.17 ± 0.12% in atypical hyperplasias, 0.61 ± 0.88% in in situ carcinomas and 0.94 ± 1.21% in invasive carcinomas. In all cases strong caspase 3, 6 and 8 positivity was significantly associated with the extent of apoptosis ($P < 0.001$, $P = 0.015$ and $P = 0.050$ respectively). The results show that synthesis of caspases 3, 6 and 8 is up-regulated in neoplastic breast epithelial cells in parallel to the increase in the apoptotic index and progression of the breast lesions. © 1999 Cancer Research Campaign

**Keywords:** apoptosis; caspase; breast; carcinoma

Caspases are molecules involved in the terminal execution of apoptosis (Thornberry et al, 1994; Alnemri et al, 1996; Barge et al, 1997; Harvey et al, 1997; Thornberry and Lazebnik, 1998). They are activated in a cascade-like fashion and are able to cleave substrate proteins at a consensus sequence following aspartic acid residues which may, however, be different for different caspases (Patel et al, 1996; Faleiro et al, 1997; Thornberry and Lazebnik, 1998). Their substrates include DNA repair enzymes, such as poly(ADP-ribose)polymerase, several structural proteins of the cells, such as nuclear lamins, fodrin, β-catenin and cytokeratin 18, oncoproteins such as mdm2 and tumour suppressor gene products such as retinoblastoma protein (Patel et al, 1996; Rao et al, 1996; Brancolini et al, 1997; Caulin et al, 1997; Chen et al, 1997; Tan et al, 1997). Caspases are also able to activate DNAase and are thus required for the typical DNA fragmentation found in apoptosis (Enari et al, 1998; Janicke et al, 1998). They are resident proteins of the cytosol and when activated, the aminoterminal fragment of the molecule is removed and the rest of the molecule is cleaved into 10- and 20-kDa fragments, which form an active $\alpha_2\beta_2$ tetrameric structure (Thornberry et al, 1994; Martins et al, 1997; Thornberry and Lazebnik, 1998).

There are at least 13 mammalian caspases known so far (Thornberry and Lazebnik, 1998). They can be divided in subgroups according to their phylogenetic development, structure or their order in the caspase activation cascade (Alnemri et al, 1996; Barge et al, 1997; Harvey et al, 1997). Caspases can be activated by mitochondrial substances such as cytochrome c or apoptosis-inducing factor (AIF) the release of which is induced by changes in the mitochondrial membrane permeability or electric potential (Liu et al, 1996; Kluck et al, 1997; Manon et al, 1997; Yang et al, 1997). Release of cytochrome c, for instance, first leads to activation of caspase 9 which then activates caspase 3 (Nijhawan et al, 1997). The changes in the mitochondrial membranes is influenced by members of the bcl-2 family proteins (Kluck et al, 1997; Manon et al, 1997; Yang et al, 1997). These proteins modulate the apoptotic response and they can be either pro- or antiapoptotic (Yang and Korsmeyer, 1996; Kroemer, 1997). Their structure is reminiscent of membrane pore forming proteins, such as cholera toxins, and bcl-xL and bcl-2 have been shown to inhibit release of cytochrome c or AIF from mitochondria while bax has been shown to promote it (Kluck et al, 1997; Manon et al, 1997; Minn et al, 1997; Yang et al, 1997).

The caspase cascade can also be activated directly through activation of the tumour necrosis factor (TNF) receptors such as APO-1/FAS/CD95. The APO-1/FAS/CD95 receptor is stimulated by binding of the FAS ligand (Fraser and Evan, 1996; Nagata, 1997). Upon attachment with the FAS ligand the APO-1/FAS/CD95 receptor oligomerizes, and FADD/MORT1 and FLICE (procaspase 8) become associated with it, forming the so-called death-inducing signal complex (DISC) (Muzio et al, 1996; Nagata, 1997). This is followed by activation of procaspase 8, which leads to the activation of other downstream caspases and to an apoptotic demise of the cell (Muzio et al, 1996; Nagata, 1997).

*Received 11 November 1998*
*Revised 26 February 1999*
*Accepted 10 March 1999*

*Correspondence to:* Y Soini

**Table 1** The extent of apoptosis and caspase 3, 6 and 8 immunoreactivity in hyperplasias, atypical hyperplasias, in-situ and invasive carcinomas of the breast

| Diagnosis | Apoptosis % | Caspase 3 | | | Caspase 6 | | | Caspase 8 | | |
|---|---|---|---|---|---|---|---|---|---|---|
| | | + | ++ | +++ | + | ++ | +++ | + | ++ | +++ |
| Hyperplasia | 0.14 ± 0.14 | 7 | 2 | 0 | 8 | 1 | 0 | 7 | 2 | 0 |
| Atypical hyperplasia | 0.17 ± 0.12 | 9 | 2 | 1 | 9 | 3 | 0 | 6 | 8 | 0 |
| In-situ carcinoma | 0.61 ± 0.88 | 27 | 21 | 16 | 27 | 29 | 12 | 11 | 40 | 18 |
| Comedo | 1.09 ± 1.28 | 5 | 3 | 9 | 6 | 5 | 7 | 1 | 7 | 10 |
| Solid | 0.79 ± 0.81 | 3 | 6 | 0 | 4 | 6 | 0 | 0 | 8 | 2 |
| Cribriform | 0.52 ± 0.76 | 10 | 3 | 7 | 11 | 8 | 5 | 6 | 15 | 2 |
| Papillary | 0.17 ± 0.12 | 5 | 1 | 0 | 2 | 3 | 0 | 2 | 3 | 1 |
| Lobular | 0.20 ± 0.10 | 4 | 8 | 0 | 4 | 7 | 0 | 2 | 7 | 3 |
| Invasive carcinoma | 0.94 ± 1.21 | 7 | 14 | 49 | 9 | 35 | 27 | 11 | 27 | 30 |
| Ductal | 1.00 ± 1.26 | 4 | 13 | 44 | 8 | 31 | 23 | 10 | 23 | 26 |
| Lobular | 0.54 ± 0.60 | 2 | 1 | 4 | 1 | 3 | 3 | 1 | 3 | 3 |
| Mucinous | 0.47 ± 0.79 | 1 | 0 | 1 | 0 | 1 | 1 | 0 | 1 | 1 |

+ = negative or weakly positive, ++ = moderately positive, +++ = strongly positive.

Several studies have shown that apoptosis is increased in malignant tumors (reviewed in Soini et al, 1998). In breast carcinomas, high-grade lesions display a higher apoptotic index than low-grade lesions (Lipponen et al, 1994). Apoptosis in breast carcinoma is also associated with other parameters, such as proliferation, tumour ploidy and survival and it is inversely associated with a positive oestrogen or progesterone receptor status and bcl-2 expression (Lipponen et al, 1994; Mustonen et al, 1997). The anti-apoptotic effect of a positive oestrogen receptor status is mediated through stimulation of bcl-2 mRNA synthesis by oestrogen (Wang and Phang, 1995). Decreased expression of pro-apoptotic bax is associated with a poor prognosis of the breast carcinoma patients due to development of resistance to chemotherapeutic agents (Wang and Phang, 1995).

The immunohistochemical expression and distribution of different caspases and their relation to apoptosis in breast carcinomas and preneoplastic lesions has not previously been studied. In this study we evaluated the immunohistochemical distribution of caspases 3, 6 and 8 in preneoplastic and neoplastic lesions of the breast and their relation to the apoptotic index, as determined by the TUNEL method. These three caspases were chosen because of their known central roles in apoptosis (Muzio et al, 1996; Faleiro et al, 1997).

### MATERIALS AND METHODS

### Samples

One hundred and eighty breast lesions consisting of 82 invasive carcinomas, 74 in situ carcinomas, 15 atypical hyperplasias and nine benign epithelial hyperplasias were collected from the files of the Department of Pathology, Oulu University Hospital. All material was fixed in 10% neutral formalin and embedded in paraffin. The invasive carcinomas consisted of 71 ductal, seven lobular, two mucinous, one apocrine and one tubular carcinoma. The in situ carcinomas consisted of 26 cribriform, 18 comedo, 11 solid, seven papillary and 12 lobular in situ lesions. The 15 atypical hyperplasias consisted of 13 ductal and two lobular hyperplasias. The diagnosis was based on the AFIP classification of breast tumours (Rosen and Oberman, 1992). The grades of the ductal carcinomas were evaluated according to the criteria of Bloom and Richardson, and the grades of the in situ lesions according to the criteria of Holland et al (Holland et al, 1990; Elston and Ellis, 1991). The atypical hyperplasias were defined according to Tavassoli (1992). First, the apoptotic index was determined from all these tumours. Caspase 3, 6 and 8 immunohistochemistry was performed from tumour blocks where representative areas of the breast lesions were still available after the apoptotic labelling had been performed. The number of these cases is shown in Table 1. The mean follow-up was 5.7 ± 2.9 years. There were 20 stage I, 21 stage II, 21 stage III and 11 stage IV tumours. Anti-oestrogenic treatment was given to 40 patients, 35 patients received cytostatic treatment and 30 radiation therapy in some stage of the disease.

### Immunohistochemical stainings

Polyclonal rabbit anti-human caspase-3 antibody was purchased from Pharmingen (San Diego, CA, USA). According to the manufacturer, the antibody recognizes both the unprocessed 32 kDa pro-caspase-3 molecule and the fragmented larger active 17 kDa unit. Polyclonal goat anti-human antibodies to caspase 6 (mch2) and caspase 8 (mch5) were purchased from Santa Cruz Biotechnology, Inc. (Santa Cruz, CA, USA). According to the manufacturer, the mch2 antibody recognizes amino acids 157–176 and mch5 amino acids 354–373 of the carboxy terminal part of the proteins, which both belong to the processed p20 fragment of the protein. The two antibodies thus recognize both the unprocessed and the cleaved or activated form of both caspases.

Before application of the primary antibodies, the sections were heated in a microwave oven in 10 mM citric acid monohydrate, pH 6.0, for 5–10 min. After a 60-min incubation with the primary antibody (dilution 1:500 for anti-caspase 3, 1:100 for anti-caspase 6 and 8), a biotinylated secondary anti-rabbit or anti-goat antibody (all three from Dakopatts, Copenhagen, Denmark) was applied (dilution 1:200–300) followed by the avidin–biotin–peroxidase complex (Dakopatts).

For all immunostainings, the colour was developed by diaminobenzidine, whereafter the sections were lightly counter-stained with methyl green and mounted with Eukitt (Kindler, Freiburg, Germany).

© 1999 Cancer Research Campaign 

Negative control stainings were carried out by substituting non-immune goat or rabbit serum for the primary antibodies. As a positive control for the immunostainings, a lymph node with follicular hyperplasia was used.

The intensity of the immunostainings with all the antibodies was evaluated by dividing the staining reaction in four groups:

1 = weak cytoplasmic staining intensity
2 = moderate cytoplasmic staining intensity
3 = strong cytoplasmic staining intensity
4 = very strong cytoplasmic staining intensity.

The quantity of the immunostaining was evaluated as follows:

0 = No positive immunostaining
1 = < 25% of tumor cells showing cytoplasmic positivity
2 = 25–50% of tumor cells showing cytoplasmic positivity
3 = 50–75% of tumor cells showing cytoplasmic positivity
4 = > 75% of tumor cells showing cytoplasmic positivity.

A combined score for the immunostaining, based on both qualitative and quantitative immunostaining, was composed by adding both the qualitative and quantitative score which was then divided into three groups:

+ = no or weak immunostaining; score 0–2
++ = moderate immunostaining; score 3–5
+++ = strong immunostaining; score 6–8.

For oestrogen and progesterone receptor staining, the slides were rehydrated and then microwaved in EDTA buffer first for 3 min in 99°C and then for 27 min in 85°C. The endogenous peroxidase was blocked with 0.1% hydrogen peroxide in absolute methanol for 20 min. Non-specific staining was blocked by incubating the slides in normal fetal calf serum for 20 min, followed by the primary antibody and the avidin–biotin–peroxidase complex. The slides were counterstained with methyl green, and the oestrogen and progesterone receptor status was counted as previously described (Helin et al, 1989).

### 3′-end labelling of DNA in apoptotic cells

In order to detect apoptotic cells, in situ labelling of the 3′-ends of the DNA fragments generated by apoptosis-associated endonucleases was performed using the ApopTag in situ apoptosis detection kit (Oncor, Gaithersburg, MD, USA) as previously described (Törmänen et al, 1995; Soini et al, 1996). The sections, after being dewaxed in xylene and rehydrated in ethanol, were incubated with 20 μg $ml^{-1}$ Proteinase K (Boehringer Mannheim GmbH, Mannheim, Germany) at room temperature for 15 min. The endogenous peroxidase activity was blocked by incubating the slides in 2% hydrogen peroxide in PBS, pH 7.2. The slides were then treated with terminal transferase enzyme and digoxigenin-labelled nucleotides after which anti-digoxigenin–peroxidase solution was applied on the slides. The colour was developed with diaminobenzidine after which the slides were lightly counterstained with haematoxylin. For control purposes we used tissue sections from hyperplastic lymph nodes showing an increased number of apoptotic B-cells within germinal centres and a low number of apoptotic T-cells in the interfollicular areas.

### Assessment of the apoptotic index

Cells were defined as apototic if the whole nuclear area of the cell labelled positively. Apoptotic bodies were defined as small positively labelled globular bodies in the cytoplasm of the tumour cells which could be found either singly or in groups. To estimate the apoptotic index (the percentage of apoptotic events in a given area), apoptotic cells and bodies were counted in 10 high-power fields (HPFs) and this figure was divided by the number of tumour cells in the same HPFs.

In order to test the reproducibility of estimation of apoptosis by the TUNEL method we also performed apoptosis assessment by light microscopy in invasive breast carcinoma samples. The assessment was based on the morphological criteria of apoptosis described previously (Kerr et al, 1994). The morphological apoptosis was assessed from the same tumour samples and the estimation of the apoptotic index was performed in a similar manner as with the 3′-end labelling method.

### Statistical analysis

Comparisons between groups were made using the Mann–Whitney *U*-test. The significance of associations was determined using Fisher’s exact probability test and correlation analysis. Survival was analysed by applying the Kaplan–Meier method with log-rank analysis. Probability values ≤ 0.05 were considered significant.

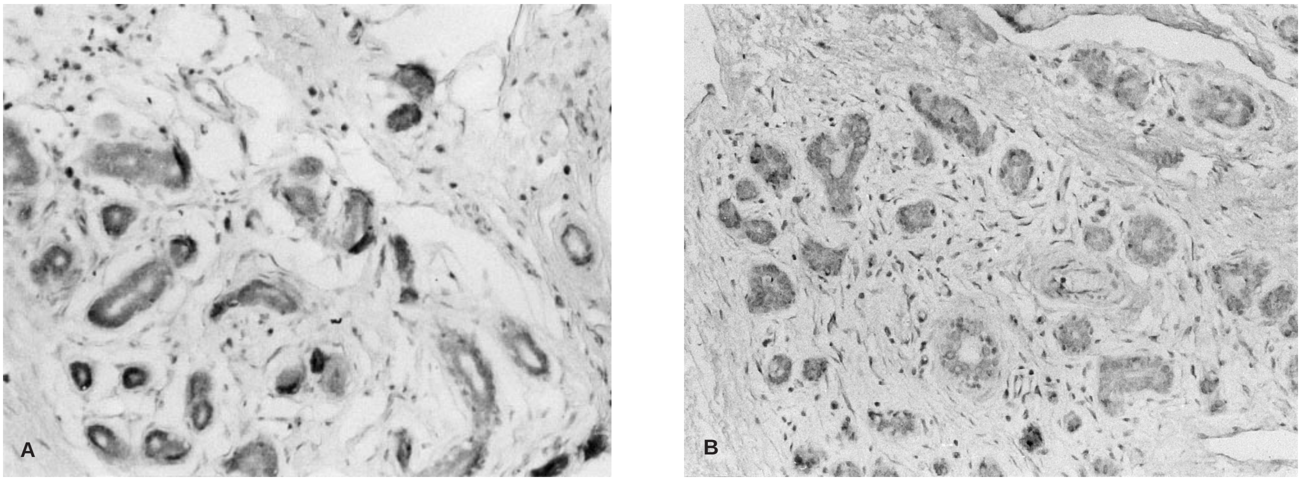

**Figure 1** Caspase immunostaining in non-neoplastic breast epithelial cells. Weak staining can be observed for caspase 3 (**A**) and caspase 8 (**B**) in the epithelial cells

*© 1999 Cancer Research Campaign*

### RESULTS

### Caspase 3, 6 and 8 expression in non-neoplastic tissues

Diffuse, weak (+) and inconsistent cytoplasmic positivity was seen for caspases 3, 6 and 8 in non-neoplastic ductal and lobular cells (Figure 1). The immunoreactivity was slightly stronger in benign epithelial lesions, like in areas with fibrocystic changes and it was also pronounced in areas with inflammation. Curiously, ducts with apocrine metaplasia frequently showed strong diffuse cytoplasmic staining for caspases, especially caspase 8.

### Caspase 3, 6 and 8 expression in breast preneoplastic and neoplastic lesions

The results of the study are compiled in Table 1. Expression of caspases 3, 6 and 8 in breast preneoplastic and neoplastic cells was mainly diffuse, intracytoplasmic (Figures 2 and 3). Caspase 6 and 8, however, also expressed granular intracytoplasmic positivity the amount of which, however, varied from tumour to tumour (Figure 4). This reaction pattern was especially prominent in invasive carcinomas and many times gave an impression of being located in the apoptotic bodies. Granular caspase 6 or 8 positivity did not, however, associate with the apoptotic index ($P = 0.19$ and $P = 0.98$ respectively). In a few cases granular staining was also seen with caspase 3. Interestingly, three carcinoma cases showed also nuclear caspase 3 staining. Nuclear staining was not seen with caspases 6 and 8. Caspase 8, on the other hand, sometimes expressed membrane-associated staining.

There was an increase in the immunoreactivity of all three caspases in parallel with the histological progression of the breast lesion. Increased caspase 3 immunopositivity (++, +++), as compared to benign ductal epithelium (+) was seen in 22% of benign epithelial hyperplasias, 25% of atypical hyperplasias, 58% of in situ carcinomas and 90% of invasive carcinomas. The corresponding percentages for caspase 6 were 11%, 25%, 60% and 87%, and for caspase 8 were 22%, 57%, 84% and 83%. There was a strong association between the expression of different caspases in atypical hyperplasias and carcinomas. Concomitant strong (+++), moderate (++), weak or negative (+) caspase 3 and 6 staining was seen in 79% of the cases ($P \leq 0.00001$). The corresponding percentages for caspase 3 and 8 and caspase 6 and 8 were 74% ($P = 0.00057$) and 71% ($P = 0.01$).

There were significantly more cases with increased (++, +++) caspase 3 immunoreactivity in invasive breast carcinomas than in in situ carcinomas ($P = 0.00002$), atypical hyperplasias ($P = 0.00001$) or benign epithelial lesions ($P = 0.00004$). Also, in-situ carcinomas had more cases with increased (++, +++) caspase 3 immunoreactivity than atypical hyperplasias ($P = 0.037$) or benign epithelial hyperplasias ($P = 0.049$). Similarly, invasive breast carcinomas had more cases showing increased (++, +++) caspase 6 positivity than in-situ carcinomas ($P = 0.00024$), atypical hyperplasias ($P = 0.00002$) or benign epithelial hyperplasias ($P = 0.00001$), and in-situ carcinomas showed more cases with increased caspase 6 immunoreactivity (++, +++) than atypical hyperplasias ($P = 0.025$) or benign epithelial hyperplasias ($P = 0.00657$). With caspase 8, however, invasive breast carcinomas did not show significantly more cases with increased (++, +++) positivity than in-situ carcinomas ($P = 0.57$) or atypical hyperplasias ($P = 0.99$), but there were more cases than in benign epithelial hyperplasias ($P = 0.00035$). Also, in-situ carcinomas did not have significantly more cases with increased (++, +++) caspase 8 positivity than atypical hyperplasias ($P = 0.99$), but the difference with benign epithelial hyperplasias was significant ($P = 0.00032$). Invasive breast carcinomas, however, showed significantly more cases with strong (+++) caspase 8 positivity than in-situ carcinomas ($P = 0.02$) or atypical hyperplasias ($P = 0.0008$), and in-situ carcinomas showed significantly more cases with strong

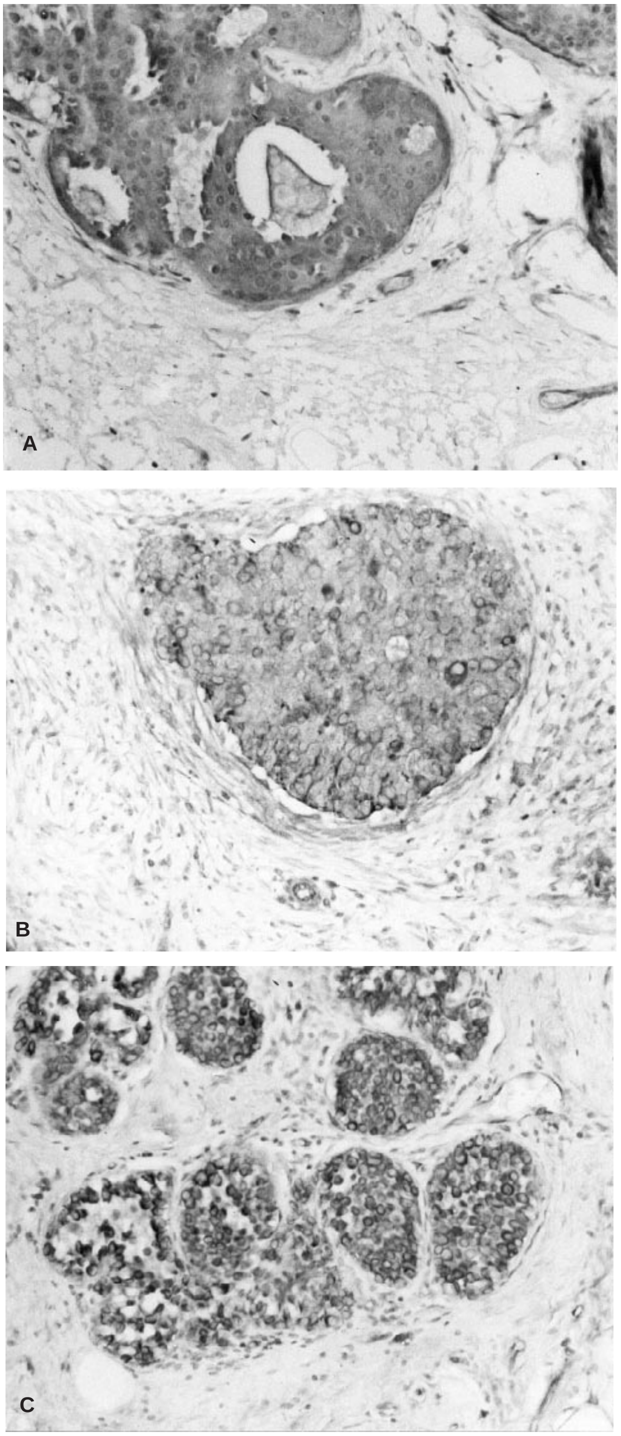

**Figure 2** Caspase staining in in situ carcinomas. Strong intracytoplasmic staining for caspase 3 (**A**) and caspase 6 (**B**) can be seen in ductal in-situ breast carcinoma and for caspase 8 (**C**) in lobular in-situ carcinoma

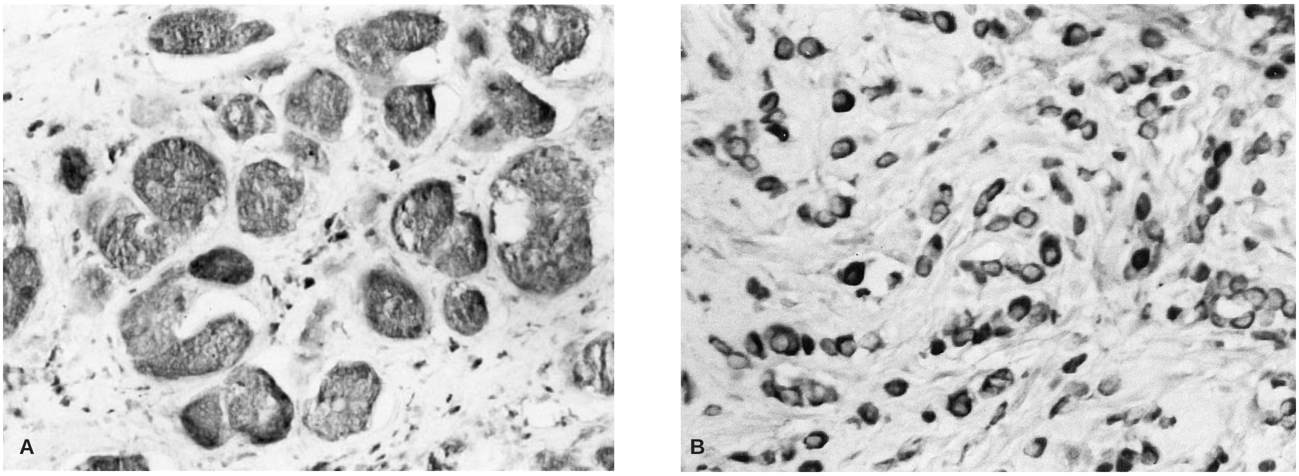

**Figure 3** Caspase staining in breast invasive carcinomas. Strong staining for caspase 3 can be seen in invasive ductal (**A**) and for caspase 6 in invasive lobular (**B**) carcinoma

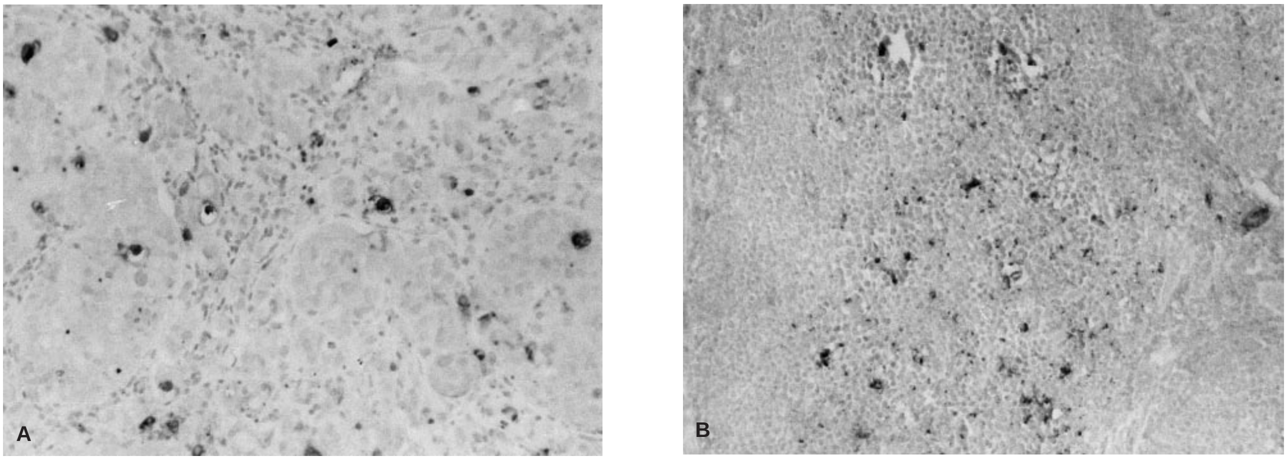

**Figure 4** Granular caspase 6 staining in invasive breast carcinoma. The black dots in the figure correspond to positively stained fragments which are reminiscent of apoptotic cells and bodies (**A**). In this figure caspase 6 immunostaining in a lymphatic follicle from the control lymph node is shown. Apoptotic cells and fragments show a similar positivity as shown in the breast carcinoma (**B**)

(+++) caspase 8-positive cases than atypical hyperplasias ($P = 0.02$). Similar associations were found with caspase 3 and 6 ($P < 0.00001$, $P = 0.00008$, $P = 0.057$ for caspase 3 respectively, and $P = 0.006$, $P = 0.00002$ and $P = 0.12$ for caspase 6 respectively).

Low- and intermediate-grade in-situ lesions of the breast had significantly fewer cases with strong (+++) caspase 3, 6 and 8 immunoreactivity than high-grade lesions ($P = 0.0045$, $P = 0.049$, $P = 0.0001$ respectively). In invasive carcinomas, however, a similar association between strong (+++) caspase immunoreactivity and high-grade tumours was not found ($P = 0.27$, $P = 0.26$ and $P = 0.69$ respectively).

### Apoptotic index in invasive and in-situ carcinomas and in atypical and benign epithelial hyperplasias

The apoptotic index in benign epithelial hyperplasias, atypical hyperplasias, in situ carcinomas and invasive carcinomas are shown in Table 1. There was a significantly lower apoptotic index in benign and atypical hyperplasias compared to in situ or invasive carcinomas ($P < 0.001$). Invasive carcinomas displayed a higher apoptotic index than atypical ($P < 0.012$) or benign epithelial hyperplasias ($P < 0.001$). Also, in situ carcinomas had a higher apoptotic index than atypical ($P = 0.004$) or benign epithelial hyperplasias ($P < 0.001$). No difference was, however, observed in the apoptotic index between atypical and benign epithelial hyperplasias ($P = 0.97$).

Low-grade in situ lesions showed a significantly lower extent of apoptosis (0.23 ± 0.20%) than intermediate- and high-grade lesions (1.05 ± 1.15%) ($P < 0.001$). Similarly, low- and intermediate-grade lesions showed a significantly lower extent of apoptosis (0.51 ± 0.73%) than high-grade lesions (1.04 ± 1.25%) ($P = 0.012$). In situ lobular carcinomas showed a significantly lower extent of apoptosis than comedo-type lesions ($P < 0.001$). The apoptotic index in grade I invasive tumours was lower (0.75 ± 0.71%) than in grade II (0.95 ± 1.55%) or grade III (1.10 ± 1.10%) lesions and lobular invasive carcinomas had a lower apoptotic index (0.54 ± 0.60%) than ductal carcinomas (1.00 ± 1.26%). There was, however, no significant difference

 *© 1999 Cancer Research Campaign*

between invasive grade I and grade II–III or grade I–II and grade III tumours ($P$ = 0.42 and $P$ = 0.22 respectively) or between invasive ductal or lobular carcinomas ($P$ = 0.97).

### Association of caspases 3, 6 and 8 with apoptosis, oestrogen and progesterone receptor status and survival

In all cases, strong (+++) caspase 3 positivity was significantly associated with increased apoptosis (1.00 ± 1.27% for strong expression, 0.36 ± 0.56% for other cases, $P$ = 0.007). A similar association was seen with caspase 6 (1.11 ± 1.21% vs 0.62 ± 0.99%, $P$ = 0.012) and caspase 8 (1.06 ± 1.24% vs 0.53 ± 0.90%, $P$ = 0.009). There was no significant association between strong (+++) caspase 3, 6 or 8 immunoreactivity and a positive oestrogen or progesterone receptor status in invasive carcinomas ($P$ = 0.29, $P$ = 0.53, $P$ = 0.54; and $P$ = 0.33, $P$ = 0.45 and $P$ = 0.08 respectively). In invasive carcinomas, no association with patient survival was observed with caspase 3, 6 or 8 ($P$ = 0.77, $P$ = 0.94 and $P$ = 0.14 respectively).

### Comparison of the TUNEL method with light microscopy

To study the reproducibility of the TUNEL method, we also evaluated the apoptotic index by morphological criteria in invasive breast carcinomas and compared the values with those obtained by TUNEL. The average apoptotic index in invasive carcinomas was 0.82 ± 0.97%. According to the results, there was a strong positive correlation between the extent of apoptosis obtained by TUNEL and light microscopic morphology (r = 0.5018, $P$ < 0.001).

### DISCUSSION

This study was undertaken to investigate the expression of caspases 3, 6 and 8 in benign and atypical hyperplasias, and in situ and invasive carcinomas of the breast. Also, the extent of apoptosis was studied and the results were correlated with the expression of the caspases in the lesions.

According to the results there was a gradual increase in the expression of caspases 3, 6 and 8 along with the progression of the breast lesion. Moreover, the expression of all these caspases was strongly associated with the rise in the apoptotic index. The results thus indicate increased synthetic activity of these proteins during neoplastic progression of breast epithelial cells. Immunohistochemistry, however, cannot discriminate between the active and inactive forms of the caspases, and only the active form is responsible for the effects of the caspases leading to apoptosis. AIF may, however, up-regulate the synthesis of caspases prior to activating them. Topoisomerase II inhibitor etoposide, for instance, up-regulates caspases 2 and 3 in neoplastic leukaemic cell lines prior to induction of apoptosis, and up-regulation of caspase 2 mRNA has been shown in response to ischaemia-induced cell death (Harvey et al, 1997; Droin et al, 1998).

In our recent study, caspase 3, 6 and 8 immunoreactivity was found in 70–80% of pancreatic adenocarcinomas (Virkajärvi et al, 1998). In lymphomas caspase 3 expression was increased in the large cell population of large- and mixed-cell follicular non-Hodgkin's lymphomas and in Reed–Sternberg cells of Hodgkin's disease (Chhanabhai et al, 1997; Krajewski et al, 1997). There may, however, be differences in the expression of caspases in histologically different tumours and tumours of different sites. In cell line studies, the expression of caspase mRNA has been shown to be heterogeneous (Droin et al, 1998). Interestingly, caspase 3 is able to modulate the function of bcl-2 by cleaving it to a shorter, truncated form which, in contrast to the longer anti-apoptotic form, is pro-apoptotic (Fujita and Tsuruo, 1998). Also, bcl-xL, another anti-apoptotic protein, is cleaved by caspases to a similar truncated pro-apoptotic fragment (Clem et al, 1998).

The immunostaining with all three caspase antibodies was cytoplasmic and mainly diffuse. This is in line with their known cytoplasmic location. Caspases 6 and 8 also showed granular or fragmented cytoplasmic staining. Such staining was only rarely observed with caspase 3. The granular or fragmented cytoplasmic staining many times appeared to result from staining of the fragments suggesting that the concentration of caspases 6 and 8 would be higher in them. The quantity of the granular staining of these two caspases did not, however, associate with the apoptotic index of breast tumours. This might be due to their heterogenous expression in apoptotic fragments in different tumours, i.e. not all tumours showed staining of apoptotic fragments for caspase 6 to 8 to a similar degree.

In addition to cytoplasmic staining, caspase 8 sometimes also expressed membrane-bound staining. Such staining might be associated with FLICE, the proform of caspase 8, and thus indirectly might reflect a high concentration of the APO-1/FAS receptor complex on the cell membrane and a consequent formation of DISC on tumour cells in some cases. Also nuclear staining was seen, but only with caspase 3. Nuclear caspase 3 staining has previously been described in some non-neoplastic tissues such as type II pneumocytes of the lung, or epithelial cells of the colon or stomach (Krajewska et al, 1997). In non-neoplastic breast epithelial cells, no nuclear staining was, however, observed. We also found a strong association between the expression of all three caspases. This might suggest a mutual regulation of their synthesis in neoplastic breast epithelial cells.

Analysis of the apoptotic index in the breast lesions showed a progressive increase in apoptosis in parallel with the biological aggressiveness of the lesion. The results further show that in in situ lesions, there are differences in the apoptotic index depending on the histology or grade of the lesions. Not surprisingly, in situ comedo-type of lesions showed the highest extent of apoptosis followed by solid, cribriform and papillary variants. The lowest apoptotic index was seen in lobular in situ carcinoma. Similarly, high-grade in situ lesions showed a higher extent of apoptosis than intermediate- or low-grade lesions. The results thus suggest that genes that are activated in high-grade lesions may also be responsible for the induction of a higher extent of apoptosis in them. The expression of some cancer genes, such as *p53* and *c-erbB2*, has been shown to be different in high-grade in situ lesions compared to low- or intermediate-grade lesions (van de Vijver et al, 1988; O'Malley et al, 1994; Mack et al, 1997). Moreover, comedo-type of in situ carcinomas display less receptor positivity than other in situ carcinomas (Bur et al, 1992; Barnes and Masood, 1990). Oestrogenic stimulation has been shown to inhibit apoptosis through up-regulation of bcl-2 mRNA, and a lack of oestrogen receptors might thus lead to an increased apoptosis in high-grade in situ lesions (Wang and Phang, 1995). In line with this, bcl-2 immunohistochemistry was shown to be reduced in poorly differentiated in situ carcinomas (Mustonen et al, 1997). Furthermore, there is also an inverse association between the apoptotic index and a positive oestrogen or progesterone receptor status and positive bcl-2 immunohistochemistry in invasive breast carcinomas

© 1999 Cancer Research Campaign 

(Lipponen et al, 1994; Mustonen et al, 1997). However, we did not observe any association between the expression of caspases and the oestrogen and progesterone receptor status suggesting that their synthesis is not influenced by stimulation of these receptors.

There are several factors which may hamper the use and evaluation of the results obtained by the TUNEL method (reviewed in Soini et al, 1998). Because of this we compared the results with light microscopic morphology and found a statistically significant correlation between both methods. Even though there was a statistically significant correlation, some labelling in the TUNEL method may also be due to DNA fragmentation not necessarily associated with apoptosis.

In conclusion, our results show that the expression of caspases 3, 6 and 8 is increased in parallel with the neoplastic progression of the breast lesions. Their abundant expression in pre-carcinomatous and carcinomatous breast lesions suggests an important role in the execution of apoptosis in malignant breast disease. In accordance with this, their expression was significantly associated with the overall apoptotic index of the lesions.

### ACKNOWLEDGEMENTS

This study was supported by the Finnish Cancer Societies.

### REFERENCES

Alnemri ES, Livingston DJ, Nicholson DW, Salvesen G, Thornberry NA, Wong WW and Yuan J (1996) Human ICE/CED-3 protease nomenclature. *Cell* **87**: 171

Barge RMY, Willemze R, Vandenabeele P, Fiers W and Beyaert R (1997) Differential involvement of caspases in apoptosis of myeloid leukemic cells induced by chemotherapy versus growth factor withdrawal. *FEBS Lett* **409**: 207–210

Barnes R and Masood S (1990) Potential value of hormone receptor assay in carcinoma in-situ of breast. *Am J Clin Pathol* **94**: 533–537

Brancolini C, Lazarevic D, Rodriguez J and Schneider C (1997) Dismantling cell–cell contacts during apoptosis is coupled to a caspase-dependent proteolytic cleavage of β-catenin. *J Cell Biol* **139**: 759–771

Bur ME, Zimarowski MJ, Schnitt SJ, Baker S and Lew R (1992) Estrogen receptor activity in carcinoma in-situ of the breast. *Cancer* **69**: 1174–1181

Caulín C, Salvesen GS and Oshima RG (1997) Caspase cleavage of keratin 18 and reorganization of intermediate filaments during epithelial cell apoptosis. *J Cell Biol* **138**: 1379–1394

Chen L, Marechal V, Moreau J, Levine AJ and Chen J (1997) Proteolytic cleavage of the mdm2 oncoprotein during apoptosis. *J Biol Chem* **272**: 22966–22973

Chhanabhai M, Krajewski S, Krajewska M, Wang HG, Reed JC and Gascoyne RD (1997) Immunohistochemical analysis of interleukin-1 beta-converting enzyme/Ced-3 family protease, CCP32/Yama/Caspase-3 in Hodgkin's disease. *Blood* **90**: 2451–2455

Clem RJ, Cheng EH, Karp CL, Kirch DG, Ueno K, Takahashi A, Kastan MB, Griffin DE, Earnshaw WC, Veliuona MA and Hardwick JM (1998) Modulation of cell death by Bcl-xL through caspase interaction. *Proc Natl Acad Sci USA* **95**: 554–559

Droin N, Dubrez L, Eymin B, Renvoize C, Breard J, Dimanche-Boitrel MT and Solary E (1998) Upregulation of CASP genes in human tumor cells undergoing etoposide-induced apoptosis. *Oncogene* **16**: 2885–2894

Elston CW and Ellis IO (1991) Pathological prognostic factors in breast cancer. I. The value of histological grade in breast cancer: experience from a large study with long term follow-up. *Histopathology* **19**: 403–410

Enari M, Sakahira H, Yokoyama H, Okawa K, Iwamatsu A and Nagata S (1998) A caspase-activated DNAase that degrades DNA during apoptosis, and its inhibitor ICAD. *Nature* **391**: 43–40

Faleiro L, Kobayashi R, Fearnhead H and Lazebnik Y (1997) Multiple species of CCP32 and Mch2 are the major active caspases present in apoptotic cells. *EMBO J* **16**: 2271–2281

Fraser A and Evan G (1996) A license to kill. *Cell* **85**: 781–784

Fujita N and Tsuruo T (1998) Involvement of bcl-2 cleavage in the acceleration of VP-16-induced U937 cell apoptosis. *Biochem Biophys Res Commun* **246**: 484–488

Harvey NL, Butt AJ and Kumar S (1997) Functional activation of Nedd2/ICH-1 (Caspase-2) is an early process in apoptosis. *J Biol Chem* **272**: 13134–13139

Helin HJ, Helle MJ, Kallioniemi O-P and Isola J (1989) Immunohistochemical determination of estrogen and progesterone receptors in human breast carcinoma. *Cancer* **63**: 1761–1767

Holland R, Hendricks JHCL, Verbeck ALM, Mravunac M and Schuurmans-Stekhoven JH (1990) Extent, distribution and mammographic/histological correlations of breast ductal carcinoma in situ. *Lancet* **335**: 519–522

Janicke RU, Sprengart ML, Wati MR and Porter AG (1998) Caspase-3 is required for DNA fragmentation and morphological changes associated with apoptosis. *J Biol Chem* **273**: 9357–9360

Kerr JFR, Winterford CM and Harmon BV (1994) Apoptosis. Its significance in cancer and cancer therapy. *Cancer* **73**: 2013–2026

Kluck RM, Bossy-Wetzel E, Green DR and Nwemeyer DD (1997) The release of cytochrome c from mitochondria: a primary site for bcl-2 regulation of apoptosis. *Science* **275**: 1132–1136

Krajewska M, Wang H-G, Krajewski S, Zapata JM, Shabaik A, Gascoyne R and Reed JC (1997) Immunohistochemical analysis of in vivo pattern of expression of CCP32 (caspase-3), a cell death protease. *Cancer Res* **57**: 1605–1613

Krajewski S, Krajewska M, Shabaik A, Miyashita T, Wang HG and Reed JC (1994) Immunohistochemical determination of in vivo distribution of bax, a dominant inhibitor of bcl-2. *Am J Pathol* **145**: 1323–133

Krajewski S, Gascoyne RD, Zapata JM, Krajewska M, Kitada S, Chhanabhai M, Horsman D, Berean K, Piro LD, Fugier Vivier I, Liu YJ, Wang HG and Reed JC (1997) Immunolocalization of the ICE/Ced-3-family protease, CPP32 (caspase-3) in non-Hodgkin's lymphomas, chronic lymphocytic leukemias, and reactive lymph nodes. *Blood* **89**: 3817–3825

Kroemer G (1997) The proto-oncogene bcl-2 and its role in regulating apoptosis. *Nat Med* **3**: 614–620

Lipponen P, Aaltomaa S, Kosma V-M and Syrjänen K (1994) Apoptosis in breast cancer as related to histopathological characteristics and prognosis. *Eur J Cancer* **30A**: 2068–2073

Liu X, Naekyung C, Yang J, Jemmerson R and Wang J (1996) Induction of apoptotic program in cell-free extracts: requirement for dATP and cytochrome c. *Cell* **86**: 147–157

Mack L, Kerkvliet N, Doig G and O'Malley FP (1997) Relationship of a new histological categorization of ductal carcinoma in situ of the breast with size and the immunohistochemical expression of p53, c-erbB2, bcl-2 and Ki-67. *Hum Pathol* **28**: 974–979

Manon S, Chaudhuri B and Guerin M (1997) Release of cytochrome c and decrease of cytochrome c oxidase in bax-expressing yeast cells, and prevention of these effects by coexpression of bcl-xL. *FEBS Lett* **415**: 29–32

Martins LM, Kottke T, Mesner PW, Basi GS, Sinha S, Frigon N, Tatar E, Tung JS, Bryant K, Takahashi A, Svingen PA, Madden BJ, McGormick DJ, Dernshaw WC and Kaufmann SH (1997) Activation of multiple interleukin-1β converting enzyme homologues in cytosol and nuclei of HL-60 cells during etoposide-induced apoptosis. *J Biol Chem* **272**: 7421–7430

Minn AJ, Velez P, Schendel SL, Liang H, Muchmore SW, Fesik SW, Fill M and Thompson CB (1997) Bcl-xL forms an ion channel in synthetic lipid membranes. *Nature* **385**: 353–357

Mustonen M, Raunio H, Pääkkö P and Soini Y (1997) The extent of apoptosis is inversely associated with bcl-2 expression in premalignant and malignant breast lesions. *Histopathology* **31**: 347–354

Muzio M, Chinnaiyan AM, Kischkel FC, O'Rourke K, Shevchenko A, Ni J, Scaffidi C, Bretz JD, Zhang M, Gentz R, Mann M, Krammer PH, Peter ME and Dixit VM (1996) FLICE, a novel FADD-homologous ICE/CED3-like protease is recruited to the CD95 (Fas/APO-1) death-inducing signaling complex. *Cell* **85**: 817–827

Nagata S (1997) Apoptosis by death factor. *Cell* **88**: 355–365

Nijhawan LP, Budihardjo I, Srinivasula SM, Ahmad M, Alnemri ES and Wang X (1997) Cytochrome c and dATP-dependent formation of Apaf-1/caspase-9 complex initiates an apoptotic protease cascade. *Cell* **91**: 479–489

O'Malley FP, Vnencak-Jones CL, Dupont WD, Parl F, Manning S and Page DL (1994) p53 mutations are confined to the comedo type ductal carcinoma in situ of the breast. Immunohistochemical and sequencing data. *Lab Invest* **71**: 67–72

Patel T, Gores GJ and Kaufmann SH (1996) The role of proteases during apoptosis. *FASEB J* **10**: 587–597

Rao L, Perez D and White E (1996) Lamin proteolysis facilitates nuclear events during apoptosis. *J Cell Biol* **135**: 1441–1455

Rosen PP and Oberman HA (1992) *Tumors of the Mammary Gland. Atlas of Tumor Pathology*. Third Series, Fascicle 7. Armed Forces Institute of Pathology: Washington, DC

 © 1999 Cancer Research Campaign

Soini Y, Virkajärvi N, Lehto V-P and Pääkkö P (1996) Hepatocellular carcinomas with a high proliferation index and a low degree of apoptosis and necrosis are associated with a shortened survival. *Br J Cancer* **73**: 1025–1030
Soini Y, Pääkkö P, Lehto V-P (1998) Histopathological evaluation of apoptosis in cancer. *Am J Pathol* **153**: 1041–1053
Tan X, Martin SJ, Green DR and Wang JYJ (1997) Degradation of retinoblastoma protein in tumor necrosis factor- and CD95-induced cell death. *J Biol Chem* **272**: 9613–9616
Tavassoli FA (1992) *Pathology of the Breast*. Appleton & Lange, Norwalk, Connecticut, USA. pp. 155–191
Thornberry NA and Lazebnik Y (1998) Caspases: enemies within. *Science* **281**: 1312–1316
Thornberry NA, Peterson EP, Zhao JJ, Howard AD, Griffin PR and Chapman KT (1994) Inactivation of interleukin-1β converting enzyme by peptide (acyloxy)methyl ketones. *Biochemistry* **33**: 3934–3940
Törmänen U, Eerola A-K, Rainio P, Vähäkangas K, Soini Y, Sormunen R, Bloigu R, Lehto V-P, Pääkkö P (1995) Enhanced apoptosis predicts shortened survival in non-small cell lung carcinoma. *Cancer Res* **55**: 5595–5602
van de Vijver MJ, Peterse JL, Mooi WJ, Wisman P, Lomans J, Dalesion O and Nusse R (1988) Neu-protein overexpression in breast cancer. Association with comedo-type ductal carcinomas in situ and limited prognostic value in stage II breast cancer. *New Engl J Med* **319**: 1239–1245
Virkajärvi N, Pääkkö P and Soini Y (1998) Apoptotic index and apoptosis influencing proteins bcl-2, mcl-1, bax and caspases 3, 6 and 8 in pancreatic carcinoma. *Histopathology* **33**: 432–439
Wang TTY and Phang JM (1995) Effects of estrogen on apoptotic pathways in human breast cancer cell line MCF-7. *Cancer Res* **35**: 2487–2489
Yang E and Korsmeyer SK (1996) Molecular thanatopsis: a discourse on the bcl-2 family and cell death. *Blood* **88**: 386–401
Yang J, Liu X, Bhalla K, Kim CN, Ibrado AM, Cai J, Peng TI, Jones DP and Wang X (1997) Prevention of apoptosis by bcl-2; release of cytochrome c from mitochondria blocked. *Science* **275**: 1129–1132

*© 1999 Cancer Research Campaign*

## References

[PDF_00512] Alnemri E. S., Livingston D. J., Nicholson D. W., Salvesen G., Thornberry N. A., Wong W. W., Yuan J. (1996). Human ICE/CED-3 protease nomenclature.. Cell.

[PDF_00515] Barge R. M., Willemze R., Vandenabeele P., Fiers W., Beyaert R. (1997). Differential involvement of caspases in apoptosis of myeloid leukemic cells induced by chemotherapy versus growth factor withdrawal.. FEBS Lett.

[PDF_00519] Barnes R., Masood S. (1990). Potential value of hormone receptor assay in carcinoma in situ of breast.. Am J Clin Pathol.

[PDF_00521] Brancolini C., Lazarevic D., Rodriguez J., Schneider C. (1997). Dismantling cell-cell contacts during apoptosis is coupled to a caspase-dependent proteolytic cleavage of beta-catenin.. J Cell Biol.

[PDF_00524] Bur M. E., Zimarowski M. J., Schnitt S. J., Baker S., Lew R. (1992). Estrogen receptor immunohistochemistry in carcinoma in situ of the breast.. Cancer.

[PDF_00526] Caulín C., Salvesen G. S., Oshima R. G. (1997). Caspase cleavage of keratin 18 and reorganization of intermediate filaments during epithelial cell apoptosis.. J Cell Biol.

[PDF_00529] Chen L., Marechal V., Moreau J., Levine A. J., Chen J. (1997). Proteolytic cleavage of the mdm2 oncoprotein during apoptosis.. J Biol Chem.

[PDF_00531] Chhanabhai M., Krajewski S., Krajewska M., Wang H. G., Reed J. C., Gascoyne R. D. (1997). Immunohistochemical analysis of interleukin-1beta-converting enzyme/Ced-3 family protease, CPP32/Yama/Caspase-3, in Hodgkin's disease.. Blood.

[PDF_00535] Clem R. J., Cheng E. H., Karp C. L., Kirsch D. G., Ueno K., Takahashi A., Kastan M. B., Griffin D. E., Earnshaw W. C., Veliuona M. A. (1998). Modulation of cell death by Bcl-XL through caspase interaction.. Proc Natl Acad Sci U S A.

[PDF_00539] Droin N., Dubrez L., Eymin B., Renvoizé C., Bréard J., Dimanche-Boitrel M. T., Solary E. (1998). Upregulation of CASP genes in human tumor cells undergoing etoposide-induced apoptosis.. Oncogene.

[PDF_00542] Elston C. W., Ellis I. O. (1991). Pathological prognostic factors in breast cancer. I. The value of histological grade in breast cancer: experience from a large study with long-term follow-up.. Histopathology.

[PDF_00545] Enari M., Sakahira H., Yokoyama H., Okawa K., Iwamatsu A., Nagata S. (1998). A caspase-activated DNase that degrades DNA during apoptosis, and its inhibitor ICAD.. Nature.

[PDF_00548] Faleiro L., Kobayashi R., Fearnhead H., Lazebnik Y. (1997). Multiple species of CPP32 and Mch2 are the major active caspases present in apoptotic cells.. EMBO J.

[PDF_00551] Fraser A., Evan G. (1996). A license to kill.. Cell.

[PDF_00552] Fujita N., Tsuruo T. (1998). Involvement of Bcl-2 cleavage in the acceleration of VP-16-induced U937 cell apoptosis.. Biochem Biophys Res Commun.

[PDF_00554] Harvey N. L., Butt A. J., Kumar S. (1997). Functional activation of Nedd2/ICH-1 (caspase-2) is an early process in apoptosis.. J Biol Chem.

[PDF_00557] Helin H. J., Helle M. J., Kallioniemi O. P., Isola J. J. (1989). Immunohistochemical determination of estrogen and progesterone receptors in human breast carcinoma. Correlation with histopathology and DNA flow cytometry.. Cancer.

[PDF_00561] Holland R., Hendriks J. H., Vebeek A. L., Mravunac M., Schuurmans Stekhoven J. H. (1990). Extent, distribution, and mammographic/histological correlations of breast ductal carcinoma in situ.. Lancet.

[PDF_00563] Jänicke R. U., Sprengart M. L., Wati M. R., Porter A. G. (1998). Caspase-3 is required for DNA fragmentation and morphological changes associated with apoptosis.. J Biol Chem.

[PDF_00566] Kerr J. F., Winterford C. M., Harmon B. V. (1994). Apoptosis. Its significance in cancer and cancer therapy.. Cancer.

[PDF_00568] Kluck R. M., Bossy-Wetzel E., Green D. R., Newmeyer D. D. (1997). The release of cytochrome c from mitochondria: a primary site for Bcl-2 regulation of apoptosis.. Science.

[PDF_00571] Krajewska M., Wang H. G., Krajewski S., Zapata J. M., Shabaik A., Gascoyne R., Reed J. C. (1997). Immunohistochemical analysis of in vivo patterns of expression of CPP32 (Caspase-3), a cell death protease.. Cancer Res.

[PDF_00578] Krajewski S., Gascoyne R. D., Zapata J. M., Krajewska M., Kitada S., Chhanabhai M., Horsman D., Berean K., Piro L. D., Fugier-Vivier I. (1997). Immunolocalization of the ICE/Ced-3-family protease, CPP32 (Caspase-3), in non-Hodgkin's lymphomas, chronic lymphocytic leukemias, and reactive lymph nodes.. Blood.

[PDF_00575] Krajewski S., Krajewska M., Shabaik A., Miyashita T., Wang H. G., Reed J. C. (1994). Immunohistochemical determination of in vivo distribution of Bax, a dominant inhibitor of Bcl-2.. Am J Pathol.

[PDF_00583] Kroemer G. (1997). The proto-oncogene Bcl-2 and its role in regulating apoptosis.. Nat Med.

[PDF_00615] Li P., Nijhawan D., Budihardjo I., Srinivasula S. M., Ahmad M., Alnemri E. S., Wang X. (1997). Cytochrome c and dATP-dependent formation of Apaf-1/caspase-9 complex initiates an apoptotic protease cascade.. Cell.

[PDF_00585] Lipponen P., Aaltomaa S., Kosma V. M., Syrjänen K. (1994). Apoptosis in breast cancer as related to histopathological characteristics and prognosis.. Eur J Cancer.

[PDF_00588] Liu X., Kim C. N., Yang J., Jemmerson R., Wang X. (1996). Induction of apoptotic program in cell-free extracts: requirement for dATP and cytochrome c.. Cell.

[PDF_00591] Mack L., Kerkvliet N., Doig G., O'Malley F. P. (1997). Relationship of a new histological categorization of ductal carcinoma in situ of the breast with size and the immunohistochemical expression of p53, c-erb B2, bcl-2, and ki-67.. Hum Pathol.

[PDF_00595] Manon S., Chaudhuri B., Guérin M. (1997). Release of cytochrome c and decrease of cytochrome c oxidase in Bax-expressing yeast cells, and prevention of these effects by coexpression of Bcl-xL.. FEBS Lett.

[PDF_00598] Martins L. M., Kottke T., Mesner P. W., Basi G. S., Sinha S., Frigon N., Tatar E., Tung J. S., Bryant K., Takahashi A. (1997). Activation of multiple interleukin-1beta converting enzyme homologues in cytosol and nuclei of HL-60 cells during etoposide-induced apoptosis.. J Biol Chem.

[PDF_00603] Minn A. J., Vélez P., Schendel S. L., Liang H., Muchmore S. W., Fesik S. W., Fill M., Thompson C. B. (1997). Bcl-x(L) forms an ion channel in synthetic lipid membranes.. Nature.

[PDF_00606] Mustonen M., Raunio H., Päkkö P., Soini Y. (1997). The extent of apoptosis is inversely associated with bcl-2 expression in premalignant and malignant breast lesions.. Histopathology.

[PDF_00609] Muzio M., Chinnaiyan A. M., Kischkel F. C., O'Rourke K., Shevchenko A., Ni J., Scaffidi C., Bretz J. D., Zhang M., Gentz R. (1996). FLICE, a novel FADD-homologous ICE/CED-3-like protease, is recruited to the CD95 (Fas/APO-1) death--inducing signaling complex.. Cell.

[PDF_00614] Nagata S. (1997). Apoptosis by death factor.. Cell.

[PDF_00618] O'Malley F. P., Vnencak-Jones C. L., Dupont W. D., Parl F., Manning S., Page D. L. (1994). p53 mutations are confined to the comedo type ductal carcinoma in situ of the breast. Immunohistochemical and sequencing data.. Lab Invest.

[PDF_00621] Patel T., Gores G. J., Kaufmann S. H. (1996). The role of proteases during apoptosis.. FASEB J.

[PDF_00623] Rao L., Perez D., White E. (1996). Lamin proteolysis facilitates nuclear events during apoptosis.. J Cell Biol.

[PDF_00635] Soini Y., Päkkö P., Lehto V. P. (1998). Histopathological evaluation of apoptosis in cancer.. Am J Pathol.

[PDF_00632] Soini Y., Virkajärvi N., Lehto V. P., Päkkö P. (1996). Hepatocellular carcinomas with a high proliferation index and a low degree of apoptosis and necrosis are associated with a shortened survival.. Br J Cancer.

[PDF_00637] Tan X., Martin S. J., Green D. R., Wang J. Y. (1997). Degradation of retinoblastoma protein in tumor necrosis factor- and CD95-induced cell death.. J Biol Chem.

[PDF_00642] Thornberry N. A., Lazebnik Y. (1998). Caspases: enemies within.. Science.

[PDF_00644] Thornberry N. A., Peterson E. P., Zhao J. J., Howard A. D., Griffin P. R., Chapman K. T. (1994). Inactivation of interleukin-1 beta converting enzyme by peptide (acyloxy)methyl ketones.. Biochemistry.

[PDF_00647] Törmänen U., Eerola A. K., Rainio P., Vähäkangas K., Soini Y., Sormunen R., Bloigu R., Lehto V. P., Päkkö P. (1995). Enhanced apoptosis predicts shortened survival in non-small cell lung carcinoma.. Cancer Res.

[PDF_00654] Virkajärvi N., Päkkö P., Soini Y. (1998). Apoptotic index and apoptosis influencing proteins bcl-2, mcl-1, bax and caspases 3, 6 and 8 in pancreatic carcinoma.. Histopathology.

[PDF_00657] Wang T. T., Phang J. M. (1995). Effects of estrogen on apoptotic pathways in human breast cancer cell line MCF-7.. Cancer Res.

[PDF_00659] Yang E., Korsmeyer S. J. (1996). Molecular thanatopsis: a discourse on the BCL2 family and cell death.. Blood.

[PDF_00661] Yang J., Liu X., Bhalla K., Kim C. N., Ibrado A. M., Cai J., Peng T. I., Jones D. P., Wang X. (1997). Prevention of apoptosis by Bcl-2: release of cytochrome c from mitochondria blocked.. Science.

[PDF_00650] van de Vijver M. J., Peterse J. L., Mooi W. J., Wisman P., Lomans J., Dalesio O., Nusse R. (1988). Neu-protein overexpression in breast cancer. Association with comedo-type ductal carcinoma in situ and limited prognostic value in stage II breast cancer.. N Engl J Med.

